# Supplementary material for: Validity of the German version of the Stay Independent Questionnaire applied by telephone interview: A diagnostic accuracy study
Source: PLoS One. 2025 Sep 3;20(9):e0319726. doi: 10.1371/journal.pone.0319726 (PMC12407426; doi:10.1371/journal.pone.0319726)
Supplement: S1 Table — (DOCX) [file pone.0319726.s001.docx]

Table S1: Assessment instruments and questionnaires for data collection

| **Instrument** | **Abbreviation** | **Source** |
| --- | --- | --- |
| Short Physical Performance Battery | SPPB | 1, 2 |
| Timed Up & Go Test | TUG | 3 |
| Falls Efficacy Scale – International | FES-I | 4, 5 |
| Falls Risk Questionnaire (Sturzrisiko-Check) | SRC | 6, 7 |
| Sarcopenia questionnaire | SARC-F | 8, 9 |
| Grip strength measurement (Jamar dynamometer) | - | 10 |
| Bioimpedance analysis (seca mBCA 515) | BIA | 11 |
| Montreal Cognitive Assessment | MoCA | 12, 13 |
| Patient Health Questionnaire 9 | PHQ-9 | 14 |
| Longitudinal Urban Cohort Ageing Study Functional Ability Index | LUCAS FI | 15 |
| Quality of Life thermometer (QoL, from EQ-5D) | - | 16, 17 |
| Comorbidies (analogous to Charlson-Comorbidity-Index) | CCI | 18, 19 |
| Numeric Rating Scale for pain | - | - |

References:

1. Penninx BW, Ferrucci L, Leveille SG, Rantanen T, Pahor M, Guralnik JM. Lower extremity performance in nondisabled older persons as a predictor of subsequent hospitalization. J Gerontol A Biol Sci Med Sci. 2000;55: M691-697. doi:10.1093/gerona/55.11.m691

2. Guralnik JM, Simonsick EM, Ferrucci L, Glynn RJ, Berkman LF, Blazer DG, et al. A short physical performance battery assessing lower extremity function: association with self-reported disability and prediction of mortality and nursing home admission. J Gerontol. 1994;49: M85-94. doi:10.1093/geronj/49.2.m85

3. Podsiadlo D, Richardson S. The timed “Up & Go”: a test of basic functional mobility for frail elderly persons. J Am Geriatr Soc. 1991;39: 142–148. doi:10.1111/j.1532-5415.1991.tb01616.x

4. Dias N, Kempen GIJM, Todd CJ, Beyer N, Freiberger E, Piot-Ziegler C, et al. [The German version of the Falls Efficacy Scale-International Version (FES-I)]. Z Gerontol Geriatr. 2006;39: 297–300. doi:10.1007/s00391-006-0400-8

5. Kempen GIJM, Yardley L, van Haastregt JCM, Zijlstra GAR, Beyer N, Hauer K, et al. The Short FES-I: a shortened version of the falls efficacy scale-international to assess fear of falling. Age Ageing. 2008;37: 45–50. doi:10.1093/ageing/afm157

6. Anders J, Dapp U, Laub S, von Renteln-Kruse W, Juhl K. [Screening of fall risk in frail, but still independently living senior citizens]. Z Gerontol Geriatr. 2006;39: 268–276. doi:10.1007/s00391-006-0395-1

7. Anders J, Dapp U, Laub S, von Renteln-Kruse W. [Impact of fall risk and fear of falling on mobility of independently living senior citizens transitioning to frailty: screening results concerning fall prevention in the community]. Z Gerontol Geriatr. 2007;40: 255–267. doi:10.1007/s00391-007-0473-z

8. Malmstrom TK, Morley JE. SARC-F: a simple questionnaire to rapidly diagnose sarcopenia. J Am Med Dir Assoc. 2013;14: 531–532. doi:10.1016/j.jamda.2013.05.018

9. Drey M, Ferrari U, Schraml M, Kemmler W, Schoene D, Franke A, et al. German Version of SARC-F: Translation, Adaption, and Validation. J Am Med Dir Assoc. 2020;21: 747-751.e1. doi:10.1016/j.jamda.2019.12.011

10. Hamilton GF, McDonald C, Chenier TC. Measurement of grip strength: validity and reliability of the sphygmomanometer and jamar grip dynamometer. J Orthop Sports Phys Ther. 1992;16: 215–219. doi:10.2519/jospt.1992.16.5.215

11. Tinsley GM, Moore ML, Silva AM, Sardinha LB. Cross-sectional and longitudinal agreement between two multifrequency bioimpedance devices for resistance, reactance, and phase angle values. Eur J Clin Nutr. 2020;74: 900–911. doi:10.1038/s41430-019-0496-8

12. Nasreddine ZS, Phillips NA, Bédirian V, Charbonneau S, Whitehead V, Collin I, et al. The Montreal Cognitive Assessment, MoCA: a brief screening tool for mild cognitive impairment. J Am Geriatr Soc. 2005;53: 695–699. doi:10.1111/j.1532-5415.2005.53221.x

13. Thomann AE, Goettel N, Monsch RJ, Berres M, Jahn T, Steiner LA, et al. The Montreal Cognitive Assessment: Normative Data from a German-Speaking Cohort and Comparison with International Normative Samples. J Alzheimers Dis. 2018;64: 643–655. doi:10.3233/JAD-180080

14. Löwe B, Kroenke K, Herzog W, Gräfe K. Measuring depression outcome with a brief self-report instrument: sensitivity to change of the Patient Health Questionnaire (PHQ-9). J Affect Disord. 2004;81: 61–66. doi:10.1016/S0165-0327(03)00198-8

15. Dapp U, Minder CE, Anders J, Golgert S, von Renteln-Kruse W. Long-term prediction of changes in health status, frailty, nursing care and mortality in community-dwelling senior citizens—results from the Longitudinal Urban Cohort Ageing Study (LUCAS). BMC Geriatr. 2014;14: 141. doi:10.1186/1471-2318-14-141

16. Greiner W, Weijnen T, Nieuwenhuizen M, Oppe S, Badia X, Busschbach J, et al. A single European currency for EQ-5D health states. Results from a six-country study. Eur J Health Econ. 2003;4: 222–231. doi:10.1007/s10198-003-0182-5

17. Marten O, Greiner W. Feasibility properties of the EQ-5D-3L and 5L in the general population: evidence from the GP Patient Survey on the impact of age. Health Econ Rev. 2022;12: 28. doi:10.1186/s13561-022-00374-y

18. Charlson ME, Pompei P, Ales KL, MacKenzie CR. A new method of classifying prognostic comorbidity in longitudinal studies: development and validation. J Chronic Dis. 1987;40: 373–383. doi:10.1016/0021-9681(87) 90171-8

19. Quan H, Li B, Couris CM, Fushimi K, Graham P, Hider P, et al. Updating and validating the Charlson comorbidity index and score for risk adjustment in hospital discharge abstracts using data from 6 countries. Am J Epidemiol. 2011;173: 676–682. doi:10.1093/aje/kwq433
