# Supplementary material for: Validity of the German version of the Stay Independent Questionnaire applied by telephone interview: A diagnostic accuracy study
Source: PLoS One. 2025 Sep 3;20(9):e0319726. doi: 10.1371/journal.pone.0319726 (PMC12407426; doi:10.1371/journal.pone.0319726)
Supplement: S3 Table — (DOCX) [file pone.0319726.s003.docx]

Table S3: Further comorbidities apart from comorbidities of the Charlson Comorbidity Index

| **Comorbidity** | **Frequency**  *(n = 190)* | **Proportion**  *(%)* |
| --- | --- | --- |
| Hypertension | 133 | 70.0 |
| Lipid metabolism disorder | 101 | 53.2 |
| Gout | 21 | 11.1 |
| Thyroid disease | 40 | 21.1 |
| Osteoarthritis | 130 | 68.4 |
| Osteoporosis | 43 | 22.6 |
| Depression | 43 | 22.6 |
| Dizziness | 115 | 60.5 |
| Parkinson’s disease | 4 | 2.1 |
| Anemia | 20 | 10.5 |
| Visual impairment | 82 | 43.2 |
| Hearing impairment | 75 | 39.5 |
