# Supplementary material for: Validity of the German version of the Stay Independent Questionnaire applied by telephone interview: A diagnostic accuracy study
Source: PLoS One. 2025 Sep 3;20(9):e0319726. doi: 10.1371/journal.pone.0319726 (PMC12407426; doi:10.1371/journal.pone.0319726)
Supplement: S4 Table — (DOCX) [file pone.0319726.s004.docx]

Table S4: Comorbidities according to falls risk assessed by the Stay Independent Questionnaire (SIQ)

|  | **SIQ +** | | **SIQ -** | | **total** | | **p-value** |
| --- | --- | --- | --- | --- | --- | --- | --- |
| **Characteristic** | *(n = 148)* | | *(n = 42)* | | *(n = 190)* | |  |
|  | **n** | **%** | **n** | **%** | **n** | **%** |  |
| Hypertension | 107 | 72.3 | 26 | 61.9 | 133 | 70.0 | > 0.05 |
| Myocardial infarction | 10 | 6.8 | 2 | 4.8 | 12 | 6.3 | > 0.05 |
| Coronary artery disease | 30 | 20.3 | 7 | 16.7 | 37 | 19.5 | > 0.05 |
| Chronic heart failure | 37 | 25.0 | 8 | 19.1 | 45 | 23.7 | > 0.05 |
| Peripheral vascular disease | 13 | 8.8 | 4 | 9.5 | 17 | 9.0 | > 0.05 |
| Stroke | 14 | 9.5 | 1 | 2.4 | 15 | 7.9 | > 0.05 |
| Hemiplegia | 4 | 2.7 | 0 | 0 | 4 | 2.1 | > 0.05 |
| Diabetes mellitus | 25 | 16.9 | 7 | 16.7 | 32 | 16.8 | > 0.05 |
| Lipid disorders | 78 | 52.7 | 23 | 54.8 | 101 | 53.2 | > 0.05 |
| Gout | 17 | 11.5 | 4 | 9.5 | 21 | 11.1 | > 0.05 |
| Thyroid disease | 29 | 19.6 | 11 | 26.2 | 40 | 21.1 | > 0.05 |
| Chronic pulmonary disease | 33 | 22.3 | 7 | 16.7 | 40 | 21.1 | > 0.05 |
| Peptic ulcer disease | 15 | 10.1 | 7 | 16.7 | 22 | 11.6 | > 0.05 |
| Chronic hepatic failure | 3 | 2.0 | 0 | 0 | 3 | 1.6 | > 0.05 |
| Chronic kidney failure | 24 | 16.2 | 5 | 11.9 | 29 | 15.3 | > 0.05 |
| Osteoarthritis | 107 | 72.3 | 23 | 54.8 | 130 | 68.4 | < 0.05 |
| rheumatic disease | 13 | 8.8 | 1 | 2.4 | 14 | 7.4 | > 0.05 |
| Osteoporosis | 34 | 23.0 | 9 | 21.4 | 43 | 22.6 | > 0.05 |
| Cancer | 35 | 23.7 | 10 | 23.8 | 45 | 23.7 | > 0.05 |
| Tumor metastasis | 5 | 3.4 | 0 | 0 | 5 | 2.6 | > 0.05 |
| Depression | 39 | 26.4 | 4 | 9.5 | 43 | 22.6 | < 0.05 |
| Dementia | 9 | 6.1 | 1 | 2.4 | 10 | 5.3 | > 0.05 |
| Dizziness | 101 | 68.2 | 14 | 33.3 | 115 | 60.5 | < 0.001 |
| M. Parkinson | 4 | 2.7 | 0 | 0 | 4 | 2.1 | > 0.05 |
| Anemia | 19 | 12.8 | 1 | 2.4 | 20 | 10.5 | < 0.05 |
| Visual impairment | 71 | 48.0 | 11 | 26.2 | 82 | 43.2 | < 0.05 |
| Hearing impairment | 62 | 41.9 | 13 | 31.0 | 75 | 39.5 | > 0.05 |
